# Differential Roles for Snapin and Synaptotagmin in the Synaptic Vesicle Cycle

**DOI:** 10.1371/journal.pone.0057842

**Published:** 2013-02-28

**Authors:** Szi-Chieh Yu, Susan M. Klosterman, Ashley A. Martin, Elena O. Gracheva, Janet E. Richmond

**Affiliations:** 1 Biological Sciences, University of Illinois at Chicago, Chicago, Illinois, United States of America; 2 Biological and Biomedical Sciences, Yale University, New Haven, Connecticut, United States of America; Virginia Commonwealth University, United States of America

## Abstract

Evoked synaptic transmission is dependent on interactions between the calcium sensor Synaptotagmin I and the SNARE complex, comprised of Syntaxin, SNAP-25, and Synaptobrevin. Recent evidence suggests that Snapin may be an important intermediate in this process, through simultaneous interactions of Snapin dimers with SNAP-25 and Synaptotagmin. In support of this model, cultured neurons derived from embryonically lethal Snapin null mutant mice exhibit desynchronized release and a reduced readily releasable vesicle pool. Based on evidence that a dimerization-defective Snapin mutation specifically disrupts priming, Snapin is hypothesized to stabilize primed vesicles by structurally coupling Synaptotagmin and SNAP-25. To explore this model *in vivo* we examined synaptic transmission in viable, adult *C. elegans* Snapin (*snpn-1*) mutants. The kinetics of synaptic transmission were unaffected at *snpn-1* mutant neuromuscular junctions (NMJs), but the number of docked, fusion competent vesicles was significantly reduced. However, analyses of *snt-1* and *snt-1;snpn-1* double mutants suggest that the docking role of SNPN-1 is independent of Synaptotagmin. Based on these results we propose that the primary role of Snapin in *C. elegans* is to promote vesicle priming, consistent with the stabilization of SNARE complex formation through established interactions with SNAP-25 upstream of the actions of Synaptotagmin in calcium-sensing and endocytosis.

## Introduction

Neurotransmitter release is dependent on the assembly of SNARE (soluble *N*-ethylmaleimide sensitive factor adaptor protein receptor) complexes formed between the plasma membrane associated proteins, Syntaxin and SNAP-25 (synaptosomal-associated protein 25 kDa) and the vesicle-associated protein Synaptobrevin (VAMP-2) [1], [2], [3], [4]. The zippering together of the coiled-coil SNARE motifs of these three proteins is thought to bring the vesicle membrane into close apposition with the plasma membrane, developing a fusion competent state in a process known as priming [5], [6]. Based on clostridial toxin disruption as well as knockout studies, all three SNARE proteins are considered to be essential components of this minimal membrane fusion machinery [7], [8], [9].

In addition to the SNAREs, the integral synaptic vesicle protein Synaptotagmin I plays a prominent role as a Ca^2+^ sensor [10], triggering the synchronous fusion of primed vesicles in response to action potential induced Ca^2+^ entry through voltage-gated Ca^2+^ channels [11]. Two Ca^2+^ binding domains within the cytoplasmic region of Synaptotagmin, called C2A and C2B, are critical for this function and exhibit increased affinity for both the SNARE complex and membrane lipids upon calcium-binding, which together promote the exocytic event [12]. The speed and accuracy of the Synaptotagmin/SNARE complex interaction is an important determinant of exocytic response kinetics [13], [14]. Recent studies have suggested that the highly conserved protein, Snapin, may also contribute to the timing and efficacy of this process [15].

Snapin was first identified as a SNAP-25 interacting protein in a yeast two hybrid screen of human brain cDNA, and was subsequently shown to bind Synaptotagmin I [16]. By interacting with both the SNARE complex and Synaptotagmin I, Snapin dimers are postulated to promote the Synaptotagmin I/SNARE complex interaction. Phosphorylation of Snapin by protein kinase A (PKA) enhances the association of Snapin and Synaptotagmin I with the SNARE complex thus, Snapin is also a potential effector of PKA-dependent synaptic facilitation [17], [18], [19].

Although Snapin and Synaptotagmin I are predicted to act in the same pathway on the basis of biochemical interactions, analysis of vertebrate Synaptotagmin I and Snapin mutant cultured neurons reveal different phenotypes. Specifically, neurons derived from Snapin mutants have reduced synaptic event frequency [15] whereas studies of Synaptotagmin I mutants often report increased endogenous rates [20], [21], [22], although not in autaptic cultures [11], possibly reflecting differences in synaptic behavior with different culture conditions [23]. Furthermore, in some studies Synaptotagmin I has also been implicated in endocytosis, possibly by binding to the heterooligomeric AP-2 protein complex, which recruits clathrin and results in the formation of clathrin-coated vesicles [24], [25], [26], [27]. An endocytic role for Snapin is less clear, although cultured Snapin mutant neurons have fewer vesicles, which could reflect a vesicle recycling defect [15]. However, cell culture experiments such as these are not always able to fully recapitulate protein function in the intact nervous system. Neither the role of Snapin nor its functional interplay with Synaptotagmin has been examined *in vivo. C. elegans* offers an excellent model system in which to assess the consequences of mutating Snapin (*snpn-1),* Synaptotagmin (*snt-1)*, as well as double mutants, at the behavioral, electrophysiological, and ultrastructural levels within an intact organism.

## Materials and Methods

### Genetics

Nematodes were maintained on agar plates seeded with OP50 bacteria. Strains used were the wild type reference strain N2 Bristol, *snpn-1(tm1892)* 2× outcrossed, NM204 *snt-1(md290)*, SY1297 *snt-1(md290); snpn-1(tm1892)*, GH19 *glo-2(zu455)*, ZM1462 *nuIs94[Pacr-2::SNB-1::GFP]*, SY1368 *snpn-1(tm1892)*; *nuIs94[Pacr-2::SNB-1::GFP],* SY1361 *snt-1(md290)*; *[Pacr-2::SNB-1::GFP],* SY1449 *snpn-1(tm1982); jaIs1092*(integrated P*unc-17::snpn-1*; 6× outcrossed), SY1498 *jaEx1058[Psnpn-1::GFP-snpn-1::snpn-1utr(pSY1); Prab-3::mCherry::unc-54utr(pGH8)]; snpn-1(tm1892)*.

Standard conventional cloning protocols were used to generate the SNPN-1 over-expression vector for cholinergic rescue *[Punc-17::snpn-1]*. Genomic DNA for the *C. elegans snapin* gene, *snpn-1* was amplified from adult hermaphrodites using the following primers, primer 1∶5′-ACGGATCCATGTCGTCAACTGCTGGAGGCGAAGTG and primer 2∶5′-CAGGATCCGAAAATAGACAAACAGCTGCCG. The primers each contain a BamHI restriction site that was used to ligate this product into a vector containing the *Punc-17 promoter*.

The multisite gateway three-fragment vector construction protocol (Invitrogen cat. 12537-023) was used to generate the SNPN-1 expression vector, *jaEx1058[Psnpn-1::GFP-snpn-1::snpn-1utr]*. Primer 3∶5′-AAAACGTAATTGGCTGCCGATTTTGAG and primer 4∶5′-GAAAAATGAAGGAAGTTGGCTTCAGAG were used to amplify a region spanning six hundred and seventy five base pairs upstream of the *snpn-1* start codon, the *snpn-1* gene, and its 3′UTR from adult hermaphrodite genomic DNA using the HotStarTaq Plus Master Mix kit (Qiagen, cat. 203643). The PCR product was then cloned into pCRII-Blunt-TOPO vector using the Zero Blunt TOPO PCR Cloning Kit. From this PCR product the *snpn-1* promoter and the *snpn-1* coding region plus 3′UTR were separately amplified using gateway primers (primer 5∶5′-GGGGACAACTTTGTATAGAAAAGTTGCCAAAACGTAATTGGCTGCCGATT and primer 6∶5′- GGGGACTGCTTTTTTGTACAAACTTGTCATTTTAGCTGTAAGAAAGAAGA for the *snpn-1* promoter, and primer 7∶5′- GGGGACAGCTTTCTTGTACAAAGTGGCCATGTCGTCAACTGCTGGAGGCG, primer 8∶5′-GGGGACAACTTTGTATAATAAAGTTGTGAAAAATGAAGGAAGTTGGCTTC) for the *snpn-1* coding region and 3′UTR). The *snpn-1* promoter region was then cloned into the pDONR221 P4-P1r vector. The GFP sequence without a stop codon was cloned into the pDONR221 vector and the genomic *snpn-1* sequence along with 848 base pairs of downstream sequence after the stop codon was cloned into pDONR221 P2r-P3. A ligation reaction was then performed so that all three sequences in the donor vectors were in the destination vector, pDEST R4-R3 Vector II producing an N-terminally GFP-tagged SNPN-1 under the *snpn-1* promoter.

### Quantitative RT-PCR

Total mRNA was isolated from 8 plates of the worms for each strain using TRIzol (Invitrogen) extraction. Genomic DNA was removed using the TURBO-DNAFree Kit (Ambion). Reverse transcription was done from purified mRNA using the SuperScript III First-Strand Synthesis System (Invitrogen) with oligo(dT) primers. qRT-PCR was performed using fluorescent detection and quantification of SYBR green-labeled PCR product using an MJResearch Opticon2 real-time thermocycler. The cycle threshold [C(t)] value for Snapin was normalized to that of a dynamin (*dyn-1*) control using the equation: ΔC(t)sample = C(t)*snpn-1* − C(t)*dyn-1*. Normalized C(t) values for the *snpn-1* mutant (tm1892) samples were then referenced to the wild type (calibrator) to determine the relative amount of *snpn-1* mRNA using the equation: ΔΔC(t)sample = ΔC(t)sample − ΔC(t)calibrator. Primers for RT-PCR were: primer 9∶5′-CTGTGGACTTGCTCCCCTAC and primer 10∶5′-TTTTGTGAGACGTTCGAGGA.

### Behavioral Assay

Behavioral analysis was conducted on N2 and the *snpn-1(tm1892)*, *snt-1(md290)*, and *snt-1(md290); snpn-1(tm1892)* mutants. Thrashing behavior for individual worms placed in M9 medium was measured per minute over a 3 minute period. Head tap assays were performed on worms acclimated for 1 minute on seeded agar plates. A worm pick was used to gently tap the worm on the head, and the total number of elicited body bends was counted. A body bend is described as the movement in which the head of the worm completes a full sinusoid.

### Electrophysiology

The dissection and electrophysiological methods were as previously described [28], [29]. Briefly, animals were immobilized with Histoacryl Blue glue, and a lateral cuticle incision was made with a glass needle, exposing the ventral medial body wall muscles. Body wall muscle recordings were made in the whole-cell voltage-clamp configuration (holding potential, −60 mV) using an EPC-10 patch-clamp amplifier and digitized at 1 kHz. The 5 mM Ca^2+^ extracellular solution consisted of 150 mM NaCl, 5 mM KCl, 5 mM CaCl_2_, 4 mM MgCl_2_, 10 mM glucose, 5 mM sucrose, and 15 mM HEPES (pH 7.3, ∼340 mOsm), Ca^2+^ was replaced with NaCl in the 1 mM Ca^2+^extracellular solution. The patch pipette was filled with 120 mM KCl, 20 mM KOH, 4 mM MgCl_2_, 5 mM (N-tris[Hydroxymethyl] methyl-2-aminoethane-sulfonic acid), 0.25 mM CaCl_2_, 4 mM Na^2^ATP, 36 mM sucrose, and 5 mM EGTA (pH 7.2, ∼315 mOsm). Data were acquired using Pulse software (HEKA, Southboro, Massachusetts, United States) run on a Dell computer. Subsequent analysis and graphing was performed using Pulsefit (HEKA), Mini analysis (Synaptosoft Inc., Decatur, Georgia, United States) and Igor Pro (Wavemetrics, Lake Oswego, Oregon, United States).

### Confocal Imaging and Analysis

Puncta/10 µm for each strain expressing SNB-1::GFP was determined as described previously (Kim *et al* 2007). In brief, young adults for each genotype were mounted on 2% agarose pads and immobilized using 10% sodium azide (Sigma) in M9 buffer. Images were obtained with a 60× objective on an Olympus Optical FV-500 laser scanning confocal microscope. Synapses along the dorsal nerve cord were analyzed using the ‘Punctaanalyser’ program in Matlab [30].

### Electron Microscopy

N2, *snpn-1(tm1892), snt-1(md290)* and *snt-1(md290)snpn-1(tm1892)* young adult hermaphrodites for each strain were prepared for high-pressure freezing as described previously [31]. Briefly, 10–15 animals were loaded in a specimen chamber filled with *Escherichia coli* and immobilized by high-pressure freezing at −180°C under high pressure in a Bal-Tec HPM010 and moved to liquid nitrogen.

Freeze substitution was performed in a Reichert AFS machine (Leica, Oberkochen, Germany) as described previously for morphological analysis, using tannic acid (0.1%) and 0.5% gluteraldehyde fixative introduced over 96 hours, followed by 2% osmium oxide (OsO4) [32]. Fixed specimens were then embedded in Araldite 502 over 48 h period at 65°C. Serial sections were cut at a thickness of 40 nm, collected on formvar-covered, carbon-coated copper grids (EMS, FCF2010-Cu), and counterstained in 2.5% aqueous uranyl acetate for 4 min, followed by Reynolds lead citrate for 2 min. Images were obtained on a Jeol JEM-1220 (Tokyo, Japan) transmission electron microscope operating at 80 kV. Micrographs were collected using a Gatan digital camera (Pleasanton, CA) at a magnification of 100×.

Morphometric analysis of ventral nerve cord serial sections was scored blind. Images were quantified using NIH ImageJ software. A synapse was defined as a set of serial sections containing a presynaptic specialization and two flanking sections from both sides without presynaptic specialization.

### Statistical Analysis

All graphed data were plotted as mean and S.E.M, and significance was assessed using the Mann-Whitney test. Statistically significant values were: not significant p>0.05, *p≤0.05, **p≤0.01, ***p≤0.001.

## Results

**Figure 1:**
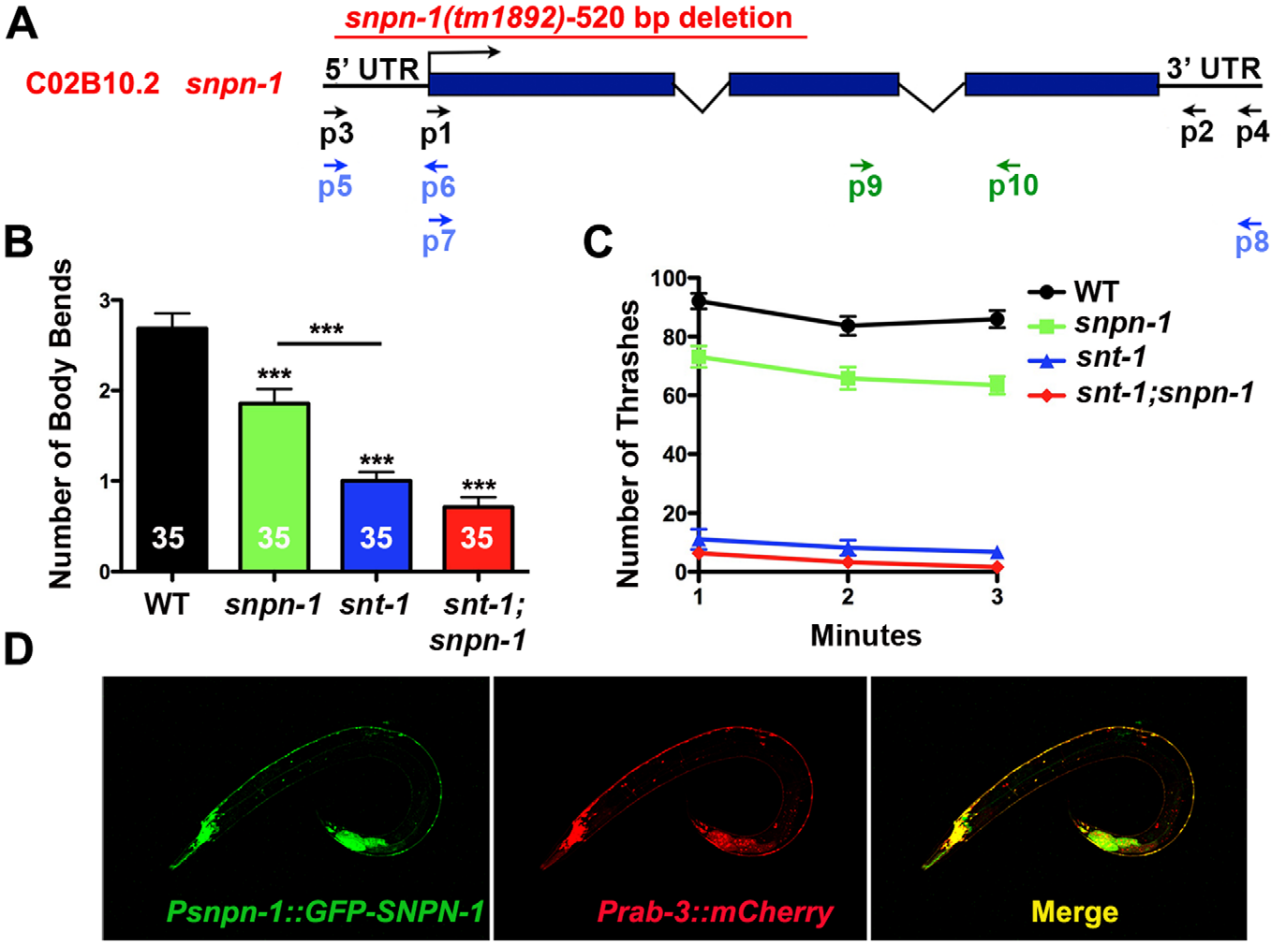
Neuronally expressed *C. elegans* Snapin and Synaptotagmin regulate locomotory behavior. (A) The Snapin (*snpn-1*) gene structure and location of the *snpn-1(tm1892)* deletion. (B) The mean ± SEM number of body bend reversals triggered by a single head tap were significantly reduced in *snpn-1(tm1892)*, *snt-1(md290)* and the double mutants. (C) The mean ± SEM values for thrashing responses of *snpn-1* mutants placed in solution show a modest decrease compared to *snt-1* and *snt-1;snpn-1* doubles mutants. It should be noted that subtle differences between *snt-1* single and double mutants would be difficult to discern in this assay, given the already very low thrashing rates observed in *snt-1* mutants. Significance values for all mutants are ≤0.0001 relative to wild-type. (D) Expression of GFP::SNPN-1 under the *snpn-1* promoter co-localized with mCherry driven by the pannueronal *pRab-3* promoter throughout the *C. elegans* nervous system.

The single *C. elegans* Snapin homolog (*snpn-1*) encodes a 122 amino acid protein that is 29% identical (59% similar) to mouse Snapin. We obtained a deletion mutant, *snpn-1(tm1892)*, which eliminates 520 genomic base pairs, spanning the upstream regulatory sequence, the start codon, the first exon and intron, and half of the second exon of the three exon *snpn-1* coding region (Fig. 1A). Based on the extent of the *snpn-1* deletion and the complete absence of a detectable transcript following qRT-PCR (data not shown), this mutant is thought to be a molecular null [33]. Unlike mouse Snapin mutants which die shortly after birth, *C. elegans snpn-1(tm1892)* mutants are viable and fertile, allowing us to assess the behavioral consequences in freely moving adult *snpn-1* nulls. A gentle tap to the head produces a reliable backing response in wild-type worms that can be scored as number of body bend reversals. As shown in Figure 1B, *snpn-1(tm1892)* mutants produced significantly fewer body bend reversals in response to a head tap when compared to wild-type worms (p = 0.0008). Similarly, the thrashing response of *snpn-1(tm1892)* mutants elicited by placing worms in M9 medium, was significantly depressed when compared to the wild type after 3 minutes (p<0.0001) (Fig. 1C). Given that the interaction between Snapin and Synaptotagmin suggests a functional link between these two proteins, we next assessed the behavioral consequences of eliminating SNPN-1 on the behavior of *C. elegans snt-1* mutants. Where as the head tap response and thrashing measurements of *snt-1(md290)* null mutants were more severely impacted in comparison to *snpn-1* mutants (Fig. 1B,C), the *snt-1;snpn-1* double mutants showed no further reduction, with the exception of the third minute of thrashing (P = 0.016) suggesting that any additive functions of SNT-1 and SNPN-1 are marginal and require prolonged activity.

The behavioral defects in *snpn-1* mutants are indicative of altered neuromuscular function. To assess whether SNPN-1 acts pre or post-synaptically at neuromuscular junctions (NMJs) we first generated an extrachromasomal GFP-tagged *snpn-1* transgene under its own promoter. GFP::SNPN-1 expression was broadly expressed in the nervous system based on colocalization with mCherry driven by the *C. elegans rab-3* promoter which is panneuronally expressed (Fig. 1D), but absent from body wall muscles suggesting that SNPN-1 plays a presynaptic role.

**Figure 2:**
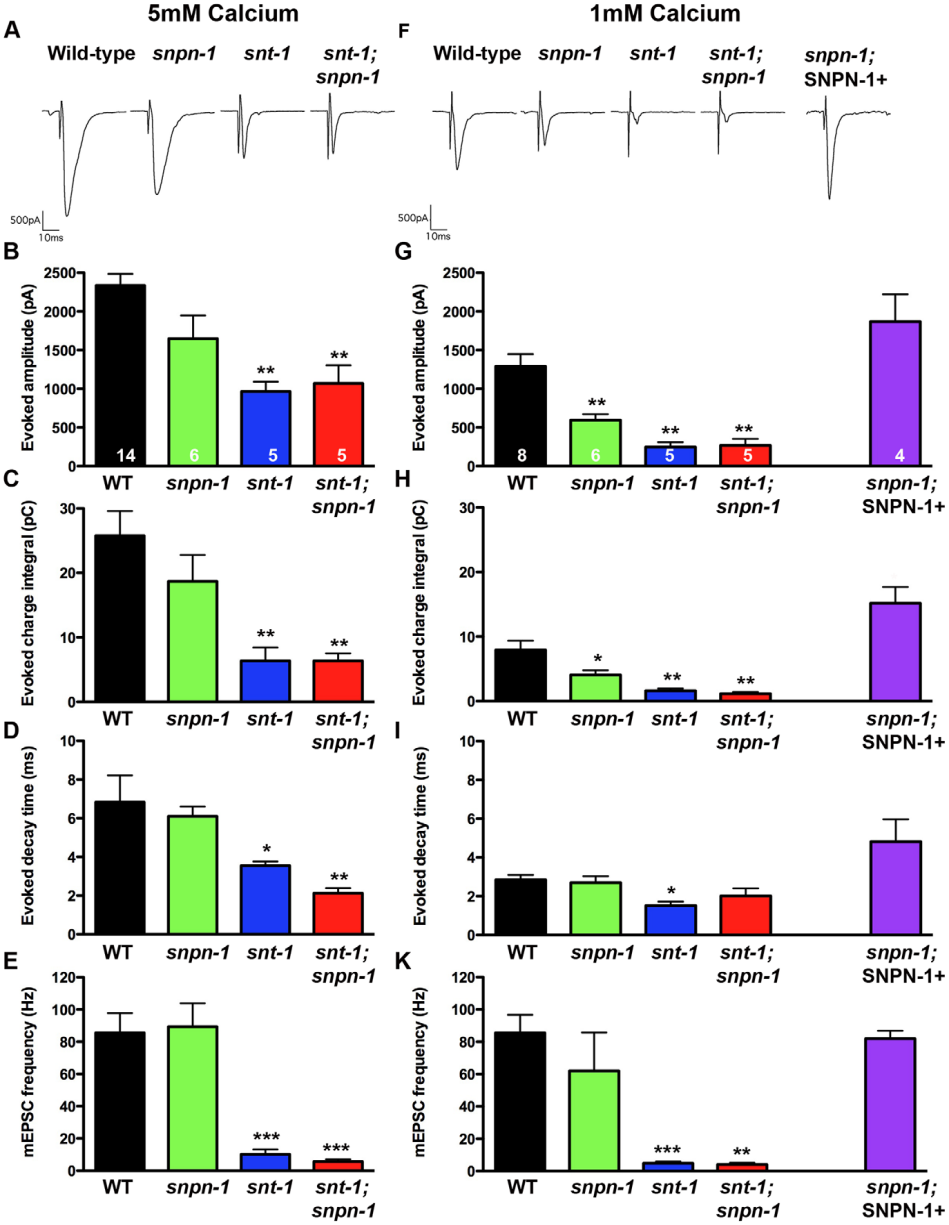
Electrophysiological analysis demonstrates synaptic defects in *snpn-1* and *snt-1* mutants. (A,F) Representative evoked post-synaptic responses from voltage-clamped body wall muscles following nerve cord stimulation in acutely dissected worms in 5 mM Ca^2+^ (A) and 1 mM Ca^2+^ (B) extracellular saline. Plots of the mean ± SEM for evoked amplitude (B,G), evoked charge integral (C,H) evoked decay (D,I) and endogenous mini frequency (E,K) demonstrate that both *snpn-1* and *snt-1* mutants have release defects that are more severe in *snt-1* mutant. The significant synaptic defects of *snpn-1* mutants observed under low 1 mM Ca^2+^ conditions are fully rescued by expressing *SNPN-1* under the cholinergic neuronal *Punc-17* promoter(F-K). In all parameters plotted, *snpn-1;snt-1* double mutants do not exhibit additivity.

To directly assay for alterations in synaptic transmission, recordings were made from the cholinergic NMJs of dissected worms. These *in situ* recordings, initially performed in the presence of 5 mM Ca^2+^ Ringer, revealed a trend toward reduced evoked junctional current (EJC) amplitudes and charge integrals in *snpn-1* mutants, although these decreases did not reach significance (p = 0.0635 and p = 0.174, respectively) (Fig. 2A–C). The frequency of endogenous synaptic events in *snpn-1* mutants was also within the wild type range (p = 0.953) (Fig. 2E). Consistent with their more severe behavioral deficits, *snt-1* mutants on the other hand, showed a significant reduction in both EJC amplitude (p = 0.0014) and charge integral (p = 0.0019), as well as endogenous event frequency in 5 mM Ca^2+^ Ringer (p = 0.0007) (Fig. 2A–C,E). Both EJCs and endogenous synaptic events in the *snt-1;snpn-1* double mutants were no more severe than *snt-1* alone, showing that in the *snt-1* background, loss of SNPN-1 has no additional effect on these release parameters (Fig. 2A–C,E).

Given the established role of Synaptotagmin as a calcium-sensor for synaptic vesicle exocytosis, and the proposed functional interaction between SNT-1 and SNPN-1, we next examined EJCs in *snpn-1* and *snt-1* mutants in reduced (1 mM) Ca^2+^ Ringer, where changes in the Ca^2+^-sensitivity of release are more apparent. Under these recording conditions, EJC amplitude and charge integral of *snpn-1* mutants were significantly reduced when compared to wild-type (p = 0.0027 and p = 0.02, respectively), although the reduction was less severe than those of *snt-1* mutants (p = 0.0016 and p = 0.0016) (Fig. 2F–H). A similar trend towards fewer endogenous minis was also observed in 1 mM Ca^2+^ Ringer (*snt-1* p = 0.0007), although this was not significant for *snpn-1* mutants (p = 0.414) (Fig. 2K). As seen in 5 mM Ca^2+^ Ringer, evoked and endogenous synaptic events of *snt-1;snpn-1* doubles were no more reduced than *snt-1* alone.

Unlike mouse cultured neurons from Snapin mutants, we saw no evidence of asynchronous release in *C. elegans snpn-1* mutants or slower EJC decay kinetics in either 5 mM or 1 mM Ca^2+^ (Fig. 2D,I). However, *snt-1* mutants, showed faster decay kinetics that reached significance in all but the low calcium double mutant. This effect could be due to more efficient acetylcholine (ACh) clearance from the cleft resulting from greatly reduced ACh release levels. Alternatively, the faster decay kinetics of *snt-1* mutants may reflect a shorter release phase, associated with the release properties of the remaining unidentified calcium-sensors in the absence of SNT-1.

To determine whether the synaptic defect of *snpn-1* mutants is due to loss of neuronal SNPN-1, we integrated a non-tagged genomic *snpn-1* transgene driven by the cholinergic neuron specific promoter, *Punc-17* into the genome of *snpn-1* mutants and assessed rescue of the cholinergic EJC in 1 mM Ca^2+^ Ringer. As shown (Fig. 2F–H), *Punc-17::snpn-1* rescued the evoked response to wild-type levels (EJC amplitude p = 0.21 relative to wild-type, EJC charge integral slightly exceeding wild-type (p = 0.028)) (Fig. 2F–H), indicating that the synaptic defect is specific to the *snpn-1* gene deletion and not a background mutation, and that SNPN-1 is required presynaptically for normal synaptic transmission.

Snapin is also known to be a component of BLOC-1 (biogenesis of lysosome-related organelle complex) [33]. To address the possibility that the synaptic phenotype of *snpn-1* mutants represents disruption of BLOC-1, we recorded the NMJ evoked responses from a mutant of the BLOC-1 protein, Pallidin(*glo-2*), in 1 mM external calcium [33]. Unlike *snpn-1* mutants, *glo-2(zu455)* mutants exhibited wild-type response amplitudes (wild-type 1291+/−159 pA, n = 8, *glo-2* 1285+/−135 pA, n = 5, p = 0.94) suggesting that the *snpn-1* synaptic phenotype is not due to disruption of BLOC-1 function.

**Figure 3:**
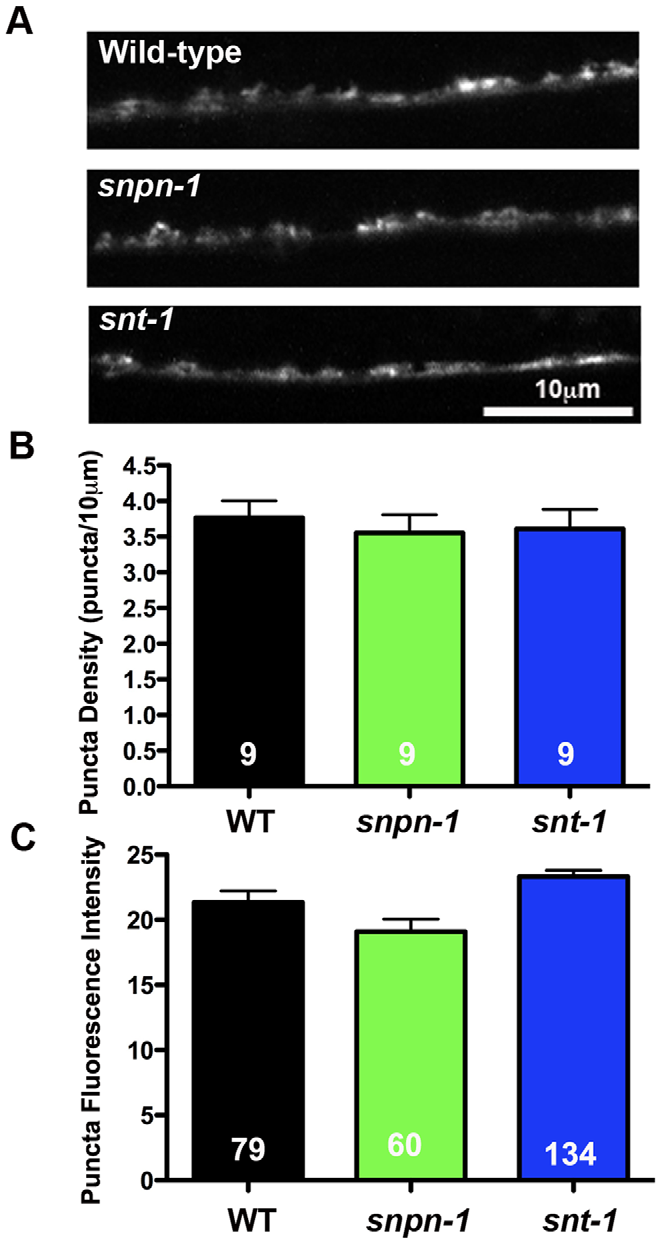
The functional defects of *snpn-1* and *snt-1* mutants are not associated with reduced synaptic density. (A) Representative confocal images of dorsal cord synaptic puncta visualized using the GFP tagged synaptic vesicle protein, Synaptobrevin (SNB-1::GFP). (B–C) Quantification of the mean ± SEM synaptic vesicle density and SNB-1::GFP puncta fluorescence show no significant differences between the *snpn-1* and *snt-1* mutants relative to wild-type.

Cultured hippocampal neurons from Snapin mutant mice have significantly fewer synapses [15]. If this phenotype is conserved, it could explain the behavioral and electrophysiological defects observed in *C. elegans snpn-1* mutants. To test whether *snpn-1* mutants have altered synaptic density, we crossed a transgenic line expressing GFP-tagged Synaptobrevin under the cholinergic neuronal promoter *Pacr-2* into the *snpn-1* mutant background. GFP puncta along the dorsal nerve cord, where individual cholinergic synapses can be readily discerned, were imaged and scored to provide a measure of synaptic density (Fig. 3). Neither the density (p = 0.51) nor average fluorescence intensity (p = 0.061) of puncta was altered in *snpn-1* mutants when compared to the wild type, indicating that the observed locomotory and electrophysiological defects were not the result of altered synaptic number (Fig. 3B,C). Similarly, *snt-1* mutants showed normal synaptic density (p = 0.57) and puncta intensity (p = 0.22) (Fig. 3B,C).

**Figure 4:**
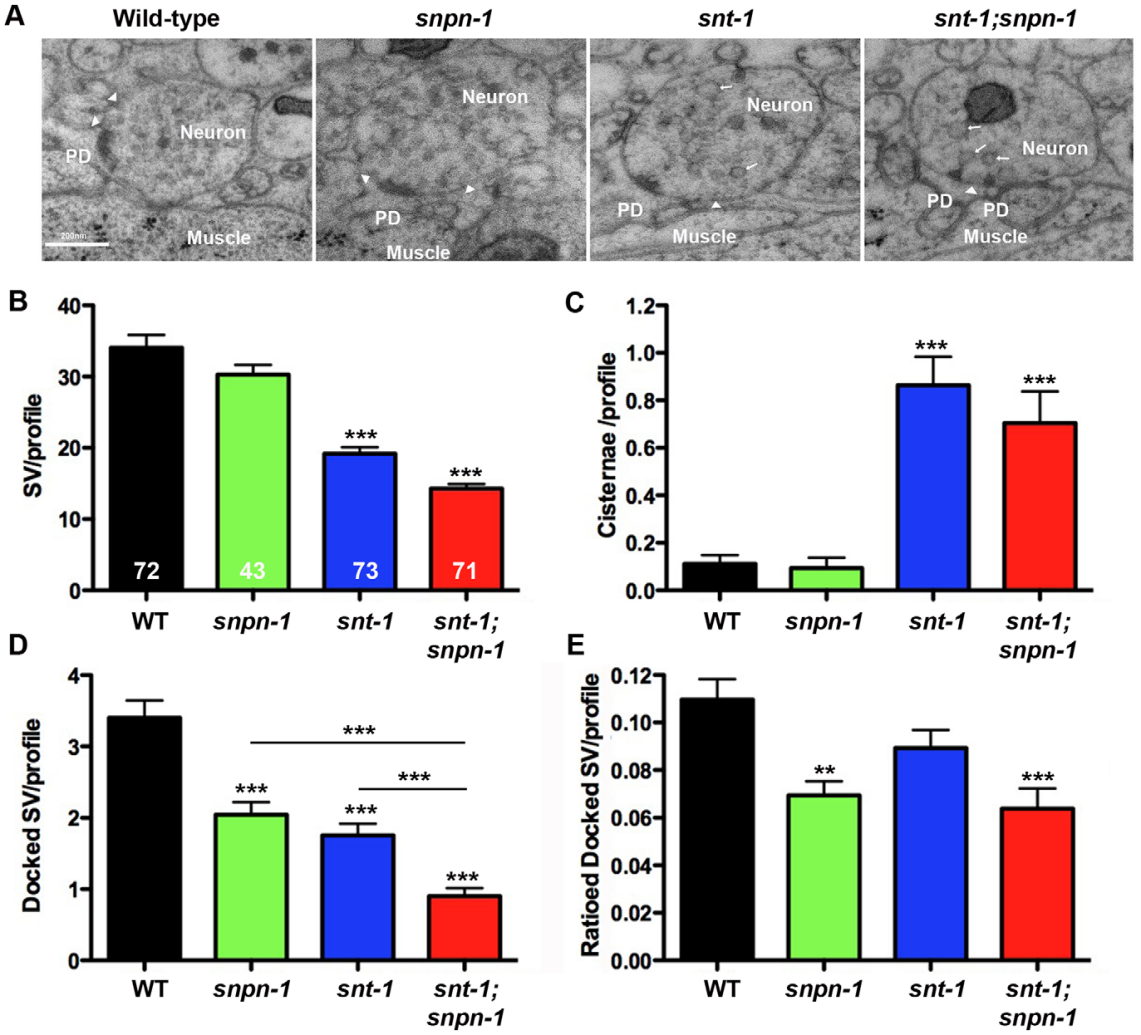
Addtive ultrastructural phenotypes of *snpn-1* and *snt-1* mutants suggest independent roles in the vesicle cycle. (A) Representative micrographs of neuromuscular junctions (NMJs) from 40 nm sections of specimens prepared by high-pressure freeze fixation (HPS). The neuronal presynaptic density in each synaptic profile is labeled PD, examples of docked synaptic vesicles (SV) are indicated with an arrowhead and cisternae are indicated by an arrow. The plotted data show that the mean ± SEM value for the number of synaptic vesicles per synaptic profile is reduced in *snt-1* but not *snpn-1* mutants (B) and that *snt-1*, but not *snpn-1* mutants exhibit significant increases in the number of cisternae, effects that are apparent in both *snt-1* single and *snt-1;snpn-1* double mutants. (C). Both *snt-1* mutant phenotypes are indicative of an endocytic defect. Plots of the number of SVs docked at the plasma membrane show reductions in both *snpn-1* and *snt-1* mutants, which are additive in the *snt-1;snpn-1* double (D), however, when the docking defect is plotted as a ratio of SVs in each profile, the docking defect only persists in the presence of the *snpn-1* mutant (E). Scale bar 200 nm.

To determine which step of the vesicle cycle is impacted in *snpn-1* mutants and to examine the degree of functional overlap between SNPN-1 and SNT-1, we prepared *snpn-1, snt-1* and *snt-1;snpn-1* double mutants for EM analysis using high-pressure freeze fixation (HPF) and freeze substitution. 40 nm serial sections were collected anterior to the vulva to obtain electron micrographs of NMJs in the region where electrophysiological recordings were made (Fig. 4A). Morphometric analyses of NMJ profiles with a visible presynaptic density were then performed. The total number of synaptic vesicles per synaptic profile was normal in *snpn-1* mutants relative to wild-type NMJs (p = 0.28), whereas the vesicle density of *snt-1* mutants was significantly reduced (p<0.0001), a phenotype that has previously been linked to an endocytic defect associated with loss of SNT-1 (Fig. 4B) [25]. The presence of large irregular cisternae in *snt-1* mutants provided further evidence of abnormal endocytosis (p<0.0001) (Fig. 4A,C). The lack of cisternae (p = 0.77 relative to wild-type) and normal vesicle density in *snpn-1* mutants suggests that SNPN-1 does not play a direct role in vesicle recycling, nor does it impact the function of SNT-1 in this process. Consistent with this conclusion *snt-1; snpn-1* double mutants have similar vesicle numbers and cisternae to the *snt-1* single mutants (Fig. 4C).

Although synaptic vesicle density was unaffected in *snpn-1* mutants, the number of vesicles that are morphologically docked on the plasma membrane was significantly reduced (p = 0.0005) (Fig. 4D). *snt-1* mutants also showed a reduction in absolute number of docked vesicles (p<0.0001) (Fig. 4D), however in the case of *snt-1*, this docking defect appeared to be a consequence of reduced vesicle density, based on the fact that the fraction of docked vesicles plotted as a function of total vesicles per profile in *snt-1* mutants was not significantly reduced compared to wild-type (p = 0.17) (Fig. 4E). The fact that the 50% reduction in absolute docked vesicles in *snt-1* mutants was less pronounced than the 75% reduction in EJC charge integral suggests that SNT-1 has additional functions beyond endocytosis, consistent with the well-documented role of *snt-1* as a calcium sensor promoting vesicle fusion. In contrast, the vesicle-docking defect of *snpn-1* mutants was not due to reduced vesicle density, and therefore implicates SNPN-1 in vesicle docking (p = 0.0016 for the fraction of docked vesicles relative to wild-type). Furthermore, the additivity of absolute docking defects observed in the *snt-1;snpn-1* double mutants (p<0.0001 relative to *snpn-1* and p = 0.0001 relative to *snt-1*), suggests that the SNPN-1 docking function is independent of SNT-1 (Fig. 4D).

**Figure 5:**
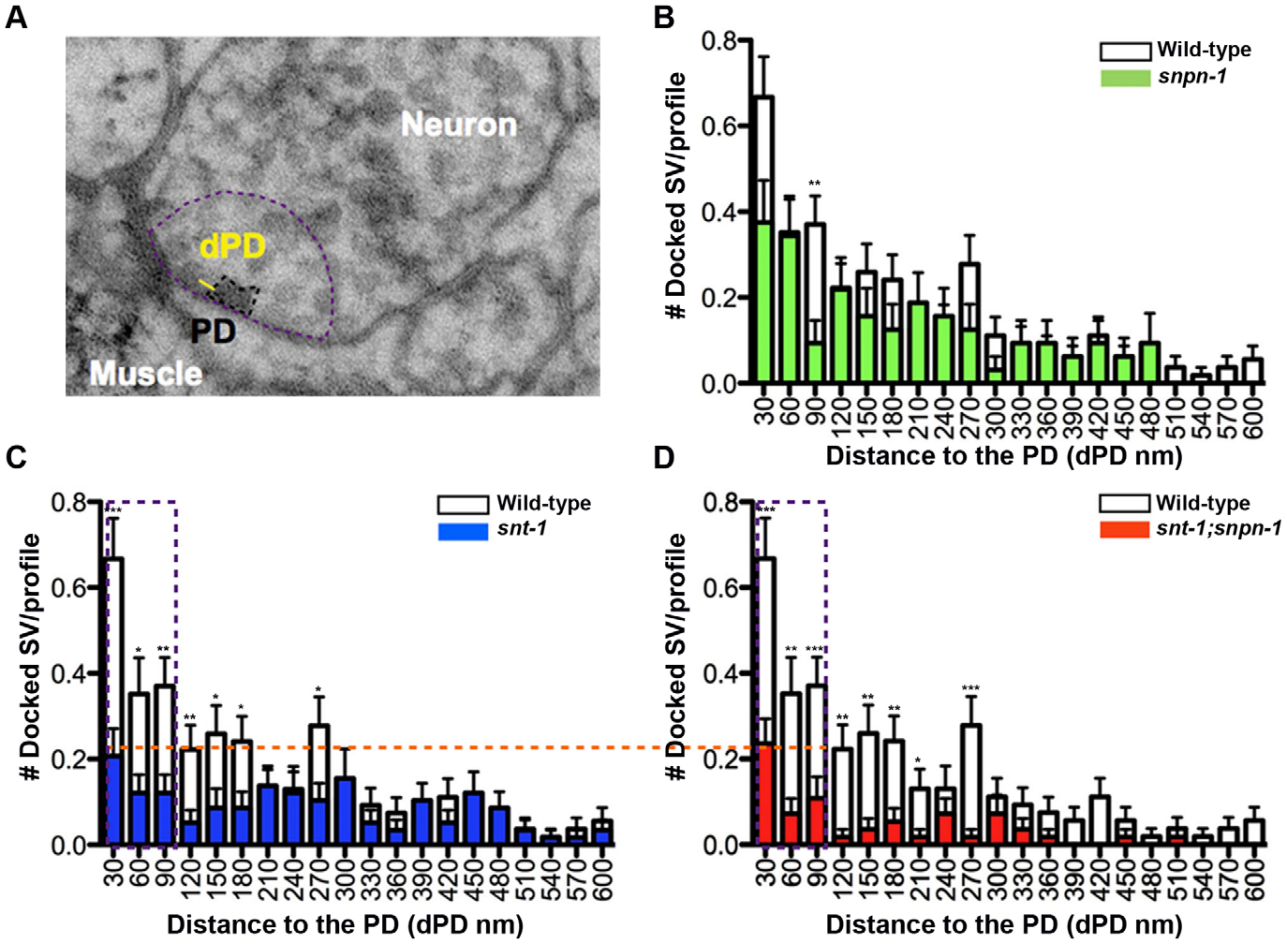
PD proximal vesicle docking deficits in *snt-1* and *snt-1;snpn-1* mutants correlate with their release defects. (A) The distance from docked synaptic vesicle (SV) membrane to the closest PD referred to as dPD, is used to plot the distribution of docked vesicles relative to the PD, in graphs (B-D). The extent of the vesicle docking defect (highlighted by the horizontal dashed line) within 90 nm of the PD (depicted as a vertical rectangular box on graphs) is similar in *snt-1* (C) and *snt-1;snpn-1* double mutants (D). All statistically significant values for dPD, plotted as mean and SEM for mutants when compared to the wild type are shown.

There is an apparent disparity between the additivity of the docking defect in the *snt-1;snpn-1* double mutant relative to the single *snt-1* mutant (p = 0.0001) (Fig. 4D), and the lack of additivity of the EJC deficit in the double mutant when compared to the *snt-1* mutant alone (EJC amplitude p = 1.0, charge integral p = 0.55) (Fig. 2G,H). To address this issue, the distribution of docked vesicles relative to the presynaptic density (Fig. 5A), the presumptive Ca^2+^ entry and release site of the NMJ, was examined in the *snpn-1* and *snt-1* single and double mutants. This analysis demonstrated that the number of vesicles docked near the presynaptic density was reduced to similar levels in *snt-1* and *snt-1;snpn-1* double mutants (Fig. 5C,D), both of which were more severe than *snpn-1* alone (Fig. 5B). Thus, the similar extent of the electrophysiological deficits observed in *snt-1* single and *snt-1;snpn-1* double mutants may be a reflection of the similar degree to which releasable docked vesicles adjacent to the presynaptic density are reduced.

## Discussion

*In situ* recordings and ultrastructural data from the NMJs of *snpn-1* mutant worms demonstrate a role for Snapin in synaptic vesicle docking and exocytosis. Specifically, *snpn-1* mutants exhibit reduced vesicle docking and a concomitant drop in evoked release amplitude, more evident in low calcium recording conditions. Cortical neurons cultured from mouse Snapin mutants also exhibit a synaptic vesicle docking defect at the EM level, however this defect is associated with a corresponding reduction in total vesicle number within these synapses which is not observed at *C. elegans snpn-1* mutant synapses [15]. Thus, the ratio of docked vesicles in mouse Snapin mutants is not impacted, suggesting that docking in mouse Snapin mutants is likely a secondary consequence of vesicle depletion.

Under the HPF fixation conditions used to prepare *C. elegans* NMJs for EM analysis, morphological vesicle docking has previously been shown to require both the priming factor UNC-13(Munc13) and the plasma membrane SNARE, UNC-64(Syntaxin), indicating that morphological docking is a correlate of vesicle priming [32], [34], [35]. A small residual docked but unprimed pool near the presynaptic density in these mutants requires interactions between vesicle-associated Rab-3(RAB-3) with integral components of the presynaptic density, Rim(UNC-10) and Liprin(SYD-2), which promote vesicle docking near release sites [34], [36], [37]. Given that vertebrate Rab-3 and Rim interact with Munc13 in a trimeric complex [38], these two processes (Rab-3/Rim dependent docking and UNC-13/SNARE-dependent priming) are thought to act in concert to maintain a superprimed vesicle pool close to the presynaptic density, the presumptive release site at which voltage gated Ca^2+^ channels are enriched. The existence of a Rab3-dependent superprimed vesicle pool was first established on the basis of recordings from mouse hippocampal neurons from Rab3 quadruple knockouts [39]. These mutants exhibit a deficit in fusion events due to loss of a subpopulation of vesicles with higher release probabilities postulated to be proximal to Ca^2+^ entry points. Consistent with this interpretation, *C. elegans rab-3*, *unc-10*(Rim) and *syd-2*(Liprin) mutants all exhibit a more pronounced reduction in evoked release under low Ca^2+^ recording conditions corresponding to loss of proximally docked vesicles [36], [37]. The observation that SNPN-1 also promotes vesicle docking near presynaptic densities could explain the increased severity of the release deficit observed in *C. elegans snpn-1* mutants under low Ca^2+^ conditions which normally favor fusion of proximal, superprimed vesicles. Given that Snapin is an established SNAP-25 binding partner, the docking defect that we observe in *C. elegans* Snapin mutants is most consistent with previous evidence implicating the Snapin/SNAP-25 interaction in the stabilization of assembled SNARE complexes, promoting priming [15], [16].

The degree to which Snapin regulates release appears to be species specific. In both *C. elegans snpn-1* mutants and a *Drosophila* Snapin RNAi line [40], the evoked release defects associated with loss of Snapin are mild. In contrast, cortical neurons derived from Snapin knockout mice, exhibit severe evoked release defects, that are still evident at synapses from heterozygotes [15]. Similarly, there are no defects in synaptic development or density in either *C. elegans* or *Drosophila* following loss of Snapin, while Snapin-null mice, die as neonates [19] and exhibit reduced brain cell density, as well as reduced cell viability and synaptic density in cultured cortical neurons [15], [41]. These mammalian neuronal growth and survival defects are associated with loss of BDNF/TrkB retrograde signaling through disruption of a Snapin/dynein interaction [41], and may exacerbate synaptic function deficits in cultured mouse Snapin mutant synapses to a greater extent than that observed *in situ* at fly and worm NMJs.

Biochemical evidence indicates that dimerized vertebrate Snapin can interact simultaneously with SNAP-25 and Synaptotagmin [15], promoting the interaction between the calcium sensor and the SNARE complex to enhance the efficacy of release of the primed vesicle pool. Consistent with this model, cultured neurons derived from Snapin mutant mice exhibit severe defects in both the frequency of endogenous release and the amplitude of evoked synaptic transmission, attributable to a reduced primed vesicle pool based on sucrose responses [15]. A similar reduction in the readily releasable pool has been observed in chromaffin cells derived from Snapin knockout mice, suggesting Snapin also promotes dense core vesicle priming [19]. Furthermore, a point mutation in vertebrate Snapin that reduces Snapin dimerization and weakens binding to both SNAP-25 and Synaptotagmin, fails to restore the vesicle priming defect of neurons from Snapin null mice [15]. While our results are consistent with a priming function for Snapin in *C. elegans*, we see no evidence that this function requires the simultaneous binding of Snapin to SNAP-25 and Synaptotagmin. Specifically, our analyses of *snt-1* and *snt-1;snpn-1* double mutants suggest that the docking defect in *C. elegans* Snapin mutants persists in the absence of Synaptotagmin. Thus in *C. elegans*, the function of Snapin in synaptic vesicle docking/priming appears to be Synaptotagmin-independent and most likely reflects disruption of Snapin interactions with SNAP-25, resulting in the destabilization of SNARE complexes. A similar conclusion was reached for the role of *Drosophila* Snapin in synaptic homeostasis, a process that exhibits genetic interactions between Snapin and SNAP-25, but is independent of Synaptotagmin [40]. It remains a possibility that the priming role of mouse Snapin is also independent of Synaptotagmin. An alternative explanation for the failure of the dimerization defective mouse Snapin mutant to rescue release amplitude could be due to the disruption of the SNAP-25 interaction rather than loss of of Synaptotagmin binding.

*C. elegans snt-1* mutants exhibit evidence of an endocytic defect, consistent with an established interaction of this protein with the AP-2 complex, required for clathrin-mediated endocytosis [24], [25], [26], [27]. Specifically, *snt-1* mutants accumulate abnormal levels of cisternae and show synaptic vesicle depletion [25]. This reduction in vesicle replenishment correlates with the pronounced reduction in endogenous mini frequency in *C. elegans snt-1* mutants, suggesting that vesicle depletion compromises endogenous release in addition to evoked release. This observation contrasts with the increase in mini frequency observed at Synaptotagmin mutant larval NMJs in *Drosophila* and in most mouse cultures, changes that have been attributed to loss of a fusion clamp normally provided by Synaptotagmin [20], [21], [22]. Possibly, the fusion clamp effect observed in these systems is masked in adult *C. elegans* as a result of a lifetime of vesicle depletion coupled with the worm’s very high endogenous release rate at NMJs, that may emphasize the endocytic defect rather than loss of a fusion clamp. In contrast to *snt-1* mutants, *C. elegans snpn-1* mutants show no evidence of a vesicle recycling defect, based on similar vesicle density and number of cisternae compared to wild-type. This again supports the conclusion of this study that the impact of Snapin on synaptic transmission in *C. elegans* appears to be independent of Synaptotagmin function.
